# Relationship between Coffee and Green Tea Consumption and All-cause Mortality in a Cohort of a Rural Japanese Population

**DOI:** 10.2188/jea.12.191

**Published:** 2007-11-30

**Authors:** Nobuo Iwai, Hitoshi Ohshiro, Youichi Kurozawa, Takenobu Hosoda, Hikari Morita, Kazuhiko Funakawa, Mikizo Okamoto, Takayuki Nose

**Affiliations:** 1 Department of Public Health, Faculty of Medicine, Tottori University.; 2 Department of Hygiene, Faculty of Medicine, Tottori University.

**Keywords:** coffee, green tea, mortality, cohort study, Japanese

## Abstract

We conducted a cohort study to investigate the effects of coffee and green tea consumption on all-cause mortality in a rural Japanese population. Data were obtained from 2,855 men and women aged 40-79 years in 1989, and during the subsequent 9.9 years of follow-up. Using the Cox regression model to adjust for potential confounding factors, we calculated the multivariate hazard ratios of death from all causes separately for men and women. The multivariate hazard ratio of mortality for men who consumed two or more cups of coffee per day, compared with those who consumed less than half a cup per day, was 0.43 (95% confidence interval, 0.30-0.63), and the ratio for those who consumed half to one cup of coffee per day was 0.70 (95% confidence interval, 0.52-0.94). Exclusion of subjects with less than 5 years of follow-up did not substantially change the findings. No other statistically significant associations were identified between consumption of the two beverages and all-cause mortality. For men, multivariate hazard ratios of death from apoplexy showed a significant inverse association with increasing coffee consumption. The effects of habitual coffee consumption and its related factors on health in Japan need to be studied in greater detail.

## INTRODUCTION

Coffee contains caffeine, which has various biological and pharmacological effects^1^^,^^2^^)^, and the relationships between coffee consumption and cardiovascular disease^3^^-^^5^^)^, cancer^6^^,^^7^^)^ and mortality^4^^,^^5^^,^^8^^-^^14^^)^ have been investigated in the United States and Europe. Green tea contains polyphenols as well as caffeine, and many investigators have examined the potential anti-carcinogenic effects of green tea^15^^-^^17^^)^. It is mostly consumed in China and Japan, and epidemiological studies on the morbidity risk associated with green tea have been naturally limited to these countries^18^^,^^19^^)^.

Over the past 50 years, the Japanese population has shifted from a traditional diet to a semi-Westernized diet^20^^)^. Coffee and green tea are the most popular beverages consumed in Japan, and coffee and green tea consumption seem indicative of Western and traditional dietary habits, respectively. Although some epidemiological studies have examined the relationship between these beverages and mortality^21^^)^ or risk of cancer (the most frequent cause of death)^22^^)^ in Japan, the extent to which coffee and green tea consumption affect the overall mortality among the general Japanese population has not been defined. We conducted a cohort study to investigate the effects of consumption of these beverages on mortality in a rural Japanese population.

## METHODS

The study subjects were residents (aged 40-79 years) of a rural community in Tottori prefecture, Japan. The study protocol was based on that of the Japan Collaborative Cohort Study for Evaluation of Cancer Risk sponsored by Monbusho (JACC Study)^23^^,^^24^^)^. Self-administered questionnaires inquiring about consumption of coffee, green tea and other foods were delivered to residents during April and May of 1989. Members of a health-promotion committee organized by the local government collected the questionnaires from the residents. A letter describing the study was also delivered to the residents and informed consent to participate in the study was obtained through their signatures on the questionnaires. Questionnaires were returned from 4,411 subjects, for a response rate of 93%.

On the questionnaire, habitual coffee and green tea consumption were simply queried by the questions “Do you drink coffee?” and “Do you drink Japanese tea (green tea)?”. The response sets for both these questions were: *almost everyday; 3-4 cups per week; 1-2 cups per week; 1-2 cups per month; seldom*. Those who answered almost everyday were asked to report the number of cups per day. We assigned a standard number of cups per day for each response. For example, the response “3-4 cups per week” was considered to be 0.5 cups per day, as is shown in Table 1. Study participants were grouped by tertiles of the distribution of coffee and green tea consumption as follows: less than 0.5 cups/day, 0.5-1 cup/day, and 2 or more cups/day for coffee; less than 0.5 cups/day, 0.5-3 cups/day, and 4 or more cups/day for green tea.

**Table 1.** The distribution of coffee and green tea consumption at baseline.

	Coffee				Green tea			
	
	Men		Women		Men		Women	

	N	%	N	%	N	%	N	%
Seldom (0 cup/day)	282	20.1	259	17.8	316	22.5	395	27.2
1-2 cups/month (0.05 cups/day)	74	5.3	58	4.0	79	5.6	88	6.1
1-2 cups/week (0.2 cups/day)	142	10.1	129	8.9	113	8.0	124	8.5
3-4 cups/week (0.5 cups/day)	148	10.5	118	8.1	143	10.2	121	8.3
Almost everyday								
1 cup/day	275	19.6	374	25.8	60	4.3	58	4.0
2-3 cups/day	421	30.0	482	33.2	326	23.2	388	26.7
4 or more cups/day	62	4.4	31	2.2	367	26.1	277	19.1

The self-administered questionnaire included histories of 10 diseases (apoplexy, hypertension, myocardial infarction, renal and liver disease, gallstone/cholecystitis, diabetes mellitus, peptic ulcer, pulmonary tuberculosis and cancer) and lifestyle factors, such as educational status, smoking, alcohol consumption and physical activities. These variables were considered as potential confounding factors.

From April 1, 1989, until December 31, 1999, death and migration from the community were determined through examining death certificates and community population registries, respectively. These follow-up data were linked with the records in the baseline questionnaire survey as well as data obtained from routine health check-ups of 1,465 subjects in 1989.

To examine the risk of death from all causes, we analyzed data from 2,855 respondents (1404 men and 1451 women) after exclusion of those for whom information about coffee and green tea consumption and confounding factors had not been obtained. During the follow-up, 361 respondents (12.6%) died (246 men and 115 women) and 130 (4.6%) moved away (55 men and 75 women). The number of years of follow-up was calculated for each respondent from April 1, 1989 until either the date of death, the date of moving or the end of the study period, whichever came first. The average follow-up period was 9.9 years. The main underlying causes of death were cancer (29%), apoplexy (17%), heart disease (15%), exogenous causes (7%), pneumonia (6%) and liver disease (5%).

### Data analysis

To examine the association between the potential confounding factors and coffee and green tea consumption, we calculated the age-adjusted proportions and mean values for each factor at each level of coffee and green tea consumption. We also calculated partial Spearman’s correlation coefficients by correlating the laboratory and physiological data with the number of cups per day of each beverage, controlling for age, among the 1,465 respondents who had received health check-ups in 1989.

Using the Cox regression model to adjust for potential confounding factors, we calculated the multivariate hazard ratios of death from all causes for each beverage consumption category separately for men and women. Besides coffee and green tea consumption (3 categories for each), the following variables were included in each model as covariates: age (in years); history of selected diseases; physical activity level (active or inactive); educational status (age at final graduation <16, <18, or >=18 years old). Additionally, the following variables were included only for men: smoking status (never smoked, smoked in the past, currently smoking =<20, or >20 cigarettes per day); habitual alcohol consumption (never drank, drank in the past, currently drinking =<46g, or >46g of ethanol). The majority of female respondents had never smoked (96%) or drunk habitually (74%). History of selected diseases included apoplexy, hypertension, myocardial infarction, liver disease, diabetes mellitus, and cancer because histories of these diseases were found to be significantly associated with all-cause mortality in the preliminary analyses. Disease histories were taken from the baseline self-administered questionnaire. However, in order to ascertain baseline cancer histories, records on the Tottori Tumor Registry were included and both the questionnaire and the tumor registry data were used. Physical activity level was categorized as active if the subject exercised or played sports at least 1 hour per week, or walked more than 1 hour per day, otherwise they were categorized as inactive. These variables, with the exception of age, were administered as dummy variables into the models.

Poor health due to a preclinical phase of cancer or due to other conditions prior to death may cause people to drink less coffee or green tea. To counter for the possibility of any such ‘preclinical effect’, we repeated the analysis after excluding respondents whose follow-up periods were less than 5 years due to death or migration. In addition, we calculated multivariate hazard ratios of death from total cancer and apoplexy for men after excluding respondents who had a history of cancer and apoplexy, respectively. Due to the small number of endpoints, this analysis was accomplished by reducing the number of covariates in the models. For the same reason, the analysis of death from other specific causes and the analysis for women could not be completed.

Multiple regression analysis and logistic regression analysis were used to analyze the trends in adjusted means and proportions, respectively. P values for trends in the Cox analysis were calculated by assigning a median value of cups per day to each category. P values less than 0.05 (two-tailed) were considered significant. All statistical analyses were performed using the Statistical Analysis System software package (SAS institute).

## RESULTS

Table 1 shows the baseline distribution of coffee and green tea consumption. Both men and women had median values of 1 cup/day of coffee, but men tended to consume more green tea (median, 1 cup/day) than women (median, 0.5 cups/day). The variation for green tea consumption was much greater than that for coffee.

Table 2 shows the age-adjusted rate of migration during the follow-up period, lifestyle characteristics and histories of diseases by beverage consumption category and sex. Persons who consumed larger amounts of coffee tended to be younger, whereas persons who consumed larger amounts of green tea tended to be older. According to the age-adjusted values, coffee consumers tended to smoke, and have lower rates of histories of several diseases such as hypertension (in men and women), liver disease, and diabetes mellitus (in men). In contrast, persons who consumed more green tea tended to have higher rates of some diseases, such as diabetes mellitus (in men), peptic ulcer, and pulmonary tuberculosis (in women). Both coffee and green tea consumption were associated with higher education in men, and with alcohol consumption in women. Women with low green tea consumption had a lower age-adjusted rate of migration than women in other categories.

**Table 2.** Potential confounding factors (age-adjusted) according to coffee and green tea consumption categories by sex.

	Coffee (cups/day)							Green tea (cups/day)						
	
	<0.5		0.5-1		>=2		Trend P*	<0.5		0.5-3		>=4		Trend P*
Men	N=498		N=423		N=483			N=508		N=529		N=367		
Migration# (%)	3.6		4.4		6.7		0.075	5.0		3.8		5.1		0.83
Age(yr):M(SD)	61.5		59.1		54.6		0.0001	56.7		57.7		61.7		0.0001
Education$ (yr):M(SE)	16.4	(9.1)	16.5	(9.4)	16.8	(9.2)	0.022	16.3	(10.1)	16.7	(9.3)	16.8	(9.0)	0.025
Currently smokes(%)	43.3	(1.0)	43.3	(1.0)	58.9	(1.0)	0.0001	47.1	(1.0)	48.8	(1.0)	50.4	(1.0)	0.64
Smoked in the past(%)	30.6		28.5		24.8		0.021	26.8		26.9		29.1		0.27
Currently drinks(%)	75.5		73.8		67.0		0.13	73.4		75.8		69.1		0.069
Drank in the past(%)	10.4		8.1		12.9		0.67	9.2		9.4		12.0		0.096
Physical inactivity(%)	32.0		33.7		36.3		0.063	35.4		33.3		31.6		0.78
*Histories of diseases*														
Apoplexy(%)	2.4		1.9		0.7		0.23	2.8		2.2		1.2		0.31
Hypertension(%)	24.1		18.2		16.3		0.0014	18.8		18.7		22.5		0.41
Myocardial infarction(%)	2.3		1.7		3.8		0.73	1.5		1.8		3.5		0.10
Renal disease(%)	4.3		4.3		3.6		0.26	4.6		1.9		5.1		0.72
Liver disease(%)	9.0		7.2		5.0		0.007	7.6		7.1		5.5		0.56
Gallstone/cholecystitis(%)	6.3		4.2		4.4		0.070	5.4		3.3		6.4		0.52
Diabetes mellitus(%)	8.9		3.4		5.9		0.012	4.6		5.1		8.5		0.024
Peptic ulcer(%)	24.7		19.7		20.8		0.068	23.5		18.0		25.1		0.35
Pulmonary tuberculosis(%)	6.7		8.3		6.2		0.76	7.1		7.9		6.2		0.88
Cancer(%)	3.4		3.0		2.0		0.37	2.2		3.9		2.6		0.68

Women	N=446		N=492		N=513			N=607		N=567		N=277		
Migration# (%)	4.8		7.5		5.3		0.92	3.4		7.3		7.1		0.041
Age(yr):M(SD)	62.2		58.3		53.9		0.0001	56.8		58.0		60.5		0.0001
Education$ (yr):M(SE)	15.9		15.9		16.1		0.072	15.9		16.1		16.0		0.48
Currently smokes(%)	1.5	(9.8)	1.1	(9.8)	6.3	(8.7)	0.0002	2.8	(9.9)	1.7	(10.1)	4.6	(9.8)	0.14
Smoked in the past(%)	1.7	(1.0)	1.3	(1.0)	1.3	(1.0)	0.92	0.8	(1.0)	1.6	(1.0)	2.7	(1.0)	0.045
Currently drinks(%)	19.9		23.0		27.7		0.001	21.9		24.3		26.6		0.031
Drank in the past(%)	2.3		1.6		2.7		0.98	1.9		2.1		2.8		0.36
Physical inactivity(%)	28.8		31.5		32.0		0.23	33.0		32.2		28.7		0.78
*Histories of diseases*														
Apoplexy(%)	1.8		0.8		1.2		0.24	1.1		1.1		2.2		0.19
Hypertension(%)	22.8		20.0		18.4		0.027	19.1		21.1		21.3		0.32
Myocardial infarction(%)	2.9		0.9		0.4		0.061	1.6		1.0		2.2		0.40
Renal disease(%)	5.6		2.7		4.5		0.68	4.0		4.2		5.1		0.55
Liver disease(%)	6.4		4.2		3.0		0.13	4.0		5.5		6.0		0.17
Gallstone/cholecystitis(%)	5.8		3.4		4.0		0.096	4.0		3.8		7.2		0.095
Diabetes mellitus(%)	3.9		2.3		1.9		0.067	3.9		2.6		1.6		0.058
Peptic ulcer(%)	12.0		7.9		9.6		0.34	8.5		9.0		12.7		0.038
Pulmonary tuberculosis(%)	4.2		4.0		2.8		0.47	2.6		4.8		4.9		0.045
Cancer(%)	1.7		2.3		2.2		0.85	2.2		1.9		2.2		0.67

* Tested using simple and multiple regression analysis for variables of age and age-adjusted values of educational status, respectively; and using logistic regression analysis for age-adjusted proportions.

# Moving away from the study community during the follow-up.

$ Age at final graduation. Tested after log-transformation.

Partial Spearman’s correlation coefficients were calculated by correlating the laboratory and physiological data with the consumption of each beverage, controlling for age, among 1,465 respondents who had received health check-ups in 1989. According to the coefficients, coffee consumption was positively correlated with serum total cholesterol in both sexes, and inversely correlated with serum aspartate aminotransferase (AST; commonly called GOT), alanine aminotransferase (ALT; commonly, GPT), and systolic and diastolic blood pressure in men in a statistically significant manner. On the other hand, green tea consumption positively correlated with either systolic or diastolic blood pressure in both sexes and with AST in women in a statistically significant manner.

Table 3 shows adjusted hazard ratios of death from all causes according to coffee and green tea consumption. The age-adjusted hazard ratios decreased significantly for men in the high and moderate coffee consumption categories. This decrease remained even after adjusting for potential confounding factors, including green tea consumption. The multivariate hazard ratio of death from all causes for men who consumed two or more cups of coffee per day, compared with those who consumed less than half a cup per day, was 0.43 (95% confidence interval, 0.30-0.63), and the ratio for those who consumed half to one cup of coffee per day was 0.70 (95% confidence interval, 0.52-0.94). The age-adjusted hazard ratio decreased significantly for women in the moderate coffee consumption category. However, after multivariate adjustment, this decrease in risk was not statistically significant nor was the decrease in risk for the high consumption category. We found no significant association between green tea consumption and all-cause mortality in both men and women. Exclusion of respondents with less than 5 years of follow-up did not substantially change the findings. The multivariate hazard ratios for men in the high and moderate coffee consumption categories were 0.34 (95% confidence interval, 0.21-0.54) and 0.67 (95% confidence interval, 0.47-0.96), respectively.

**Table 3.** Age-adjusted and multivariate hazard ratios of death from all causes according to coffee and green tea consumption.

Consumption category (cups/day)	Analysis for all respondents				Analysis after excluding respondents with less than 5 years of follow-up		
	
	No. of cases	Person- years	Age-adjusted hazard ratios (95%CI)	Multivariate hazard ratios* (95%CI)	No. of cases	Person- years	Multivariate hazard ratios* (95%CI)
Coffee							
Men							
<0.5	132	4685	1	1	89	4539	1
0.5-1	71	4162	0.71 (0.53-0.94)	0.70 (0.52-0.94)	49	4082	0.67 (0.47-0.96)
>=2	43	4858	0.50 (0.35-0.71)	0.43 (0.30-0.63)	25	4769	0.34 (0.21-0.54)
Trend P			0.0001	0.0001			0.0001

Women							
<0.5	61	4367	1	1	35	4252	1
0.5-1	31	4981	0.63 (0.41-0.98)	0.70 (0.45-1.09)	22	4911	0.88 (0.51-1.52)
>=2	23	5303	0.72 (0.44-1.20)	0.76 (0.45-1.27)	17	5258	1.00 (0.53-1.86)
Trend P			0.10	0.18			0.92


Green tea							
Men							
<0.5	85	5007	1	1	60	4916	1
0.5-3	84	5181	0.94 (0.69-1.27)	0.97 (0.71-1.31)	51	5063	0.83 (0.57-1.21)
>=4	77	3515	0.94 (0.69-1.28)	0.82 (0.60-1.12)	52	3410	0.78 (0.53-1.13)
Trend P			0.72	0.19			0.22

Women							
<0.5	50	6195	1	1	34	6130	1
0.5-3	41	5680	0.78 (0.52-1.18)	0.79 (0.52-1.20)	26	5572	0.70 (0.42-1.17)
>=4	24	2776	0.72 (0.44-1.17)	0.74 (0.45-1.21)	14	2719	0.62 (0.33-1.17)
Trend P			0.18	0.21			0.12

* Coffee and green tea consumption, age (in years), history of selected diseases, physical activity level (active or inactive), educational status (age at final graduation <16, <18, or >=18 years old), and additionally only in men, smoking status (never smoked, smoked in the past, currently smoking =<20, or >20 cigarettes per day), habitual alcohol consumption (never drank, drank in the past, currently drinking =<46g, or >46g of ethanol) were included in the Cox model as covariates.

CI= confidence interval

When we analyzed only the subjects who did not have disease histories for the 10 diseases specified in our study, the decreased risk of mortality for men with increased coffee consumption remained. The multivariate hazard ratios of death from all causes for males with no disease history in the high and moderate coffee consumption categories were 0.43 (95% confidence interval, 0.24-0.78) and 0.71 (95% confidence interval, 0.43-1.16), respectively (trend P= 0.005). In this analysis, we found no significant association between coffee consumption and mortality risk for women, nor did we find a relationship between green tea consumption and mortality risk for either men or women. We repeated the analyses after excluding subjects with less than 5 years of follow-up and obtained similar results.

Table 4 shows the multivariate hazard ratios of death for men from total cancer and apoplexy by coffee and green tea consumption category. Multivariate hazard ratios of death from apoplexy decreased significantly with increasing coffee consumption levels, while the hazard ratios of death from total cancer showed no association with coffee consumption.

**Table 4.** Multivariate hazard ratios* of death from total cancer and apoplexy according to coffee and green tea consumption for men.

Consumption category (cups/day)	Total cancer		Apoplexy	
	
	No. of cases	Multivariate hazard ratios (95%CI)	No. of cases	Multivariate hazard ratios (95%CI)
Coffee				
<0.5	24	1	22	1
0.5-1	19	0.91 (0.50-1.68)	11	0.70 (0.34-1.47)
>=2	18	0.91 (0.48-1.72)	5	0.32 (0.12-0.87)
Trend P		0.76		0.022

Green tea				
<0.5	22	1	13	1
0.5-3	21	0.93 (0.51-1.70)	15	0.96 (0.45-2.04)
>=4	18	0.92 (0.49-1.73)	10	0.60 (0.26-1.38)
Trend P		0.80		0.20

* Coffee and green tea consumption, age (in years), history of selected diseases, educational status (age at final graduation <18 or >=18 years old), and smoking status (never smoked or other status) were included in the Cox model as covariates.

CI= confidence interval

## DISCUSSION

Several lifestyle factors and disease histories are generally associated with coffee and green tea consumption^5^^,^^18^^)^. We found an association between coffee consumption and lower rates of several diseases, as well as an association between green tea consumption and higher rates of some diseases. A possible reason for these differences may be that people with a history of disease might avoid coffee, commonly believed to be a stimulant, and instead drink green tea, a beverage commonly believed to bring health benefits.

Respondents who moved away from the study community (4.6%) were lost to follow-up. However, age-adjusted rates of migration for coffee and green tea consumption groups did not differ except for slight differences seen between green tea consumption groups among women. The results from the Cox analyses did not change when we repeated the analyses after excluding the respondents who moved (data not shown).

Using the Cox multivariate model, we found a significant inverse association between coffee consumption and all-cause mortality for men, but no significant association for women. The inverse association between coffee consumption and mortality for men in the present study might be due to other dietary factors associated with coffee consumption, which could modify the risk of death. We repeated the Cox analyses thirty seven times, each time using the frequency of consumption of one of 35 different foods or the level of preference to one of two foods (oily or salty) as a covariate in the multivariate model. The decreased risk of all-cause mortality for men in the high and moderate coffee consumption categories was still observed even after considering the effects of other dietary factors. The multivariate hazard ratios of all-cause death for men in the high and moderate coffee consumption categories in these analyses ranged from 0.39 to 0.48 (the minimal lower 95% confidence limit, 0.26, and the maximal upper limits, 0.73) and from 0.64 to 0.72 (the minimal lower 95% confidence limit, 0.47, and the maximal upper limits, 0.99), respectively.

Many cohort studies have been conducted on the relationship between coffee consumption and mortality in the United States and Europe, where coffee consumption is generally higher than in Japan. Cohort studies in Finland (both men and women)^5^^)^ and Sweden (men)^8^^)^ showed a decreased risk of all-cause mortality with increased coffee consumption, whereas other studies conducted among Seventh-day Adventists^9^^)^ and company employees in the United States^10^^)^ reported an increased mortality risk for men with increased coffee consumption. No significant associations have been reported in other studies conducted in Scotland^4^^)^, Holland^12^^)^, Georgia (both men and women)^14^^)^, and California (men and women combined)^11^^)^. A weak decreased risk was observed in Norwegian men^13^^)^, but was not observed when the subjects who died within 4 years of follow-up were excluded. An association between increased coffee consumption and decreased risks of mortality from ischemic heart disease^5^^)^, apoplexy, liver cirrhosis, and suicide^11^^)^ have been reported. In contrast, other studies report an increased risk of mortality from ischemic heart disease^9^^,^^10^^)^ and suicide^25^^)^. While the conflicting findings of past studies indicate a weak association, if any, between coffee consumption and mortality, we found a strong inverse association between coffee consumption and all-cause mortality in men. Therefore, further investigation is needed to determine whether the decreased mortality observed in the present study is really due to habitual coffee consumption.

Nevertheless, our results suggest the following mechanism. According to the baseline data analysis performed on subgroups of this study, coffee consumption positively correlated with serum total cholesterol in both sexes and inversely with blood pressure and serum AST and ALT (indices of liver dysfunction) in men. Associations between habitual coffee consumption and lower blood pressure^26^^,^^27^^)^ or lower serum liver enzymes^28^^-^^30^^)^ have been reported by other investigations of different populations, including Japanese male adults, although the mechanisms behind these effects were not sufficiently studied^31^^)^. We reported in a previous study that lower serum total cholesterol, hypertension, and liver dysfunction (higher AST or ALT) were associated with an increased risk of death^32^^)^. We suggest that the inverse association between coffee and mortality observed in the present study is due to the beneficial effects of habitual coffee consumption on serum lipids, blood pressure, and liver function. The validity of this interpretation is partially supported by the results that coffee consumption was associated with decreased risk of death from apoplexy (of which hypertension and hypocholesterolemia are regarded to be risk factors in Japan). The reason why the same effects of coffee consumption on mortality, hypertension, or liver function were not observed in women is not clear. However, Tanaka et al. suggest that coffee may possibly protect against liver cell damage due to alcohol^29^^)^. According to this suggestion, the effects of coffee consumption might not be manifested in women, who generally drink less alcohol than men.

The present study also examined the relationship between green tea consumption and all-cause mortality. To our knowledge, there has been only one previous cohort study published that examined the relationship between green tea consumption and mortality in Japan^21^^)^. The previous study was conducted among female special consumers of green tea, and the results indicated that green tea was inversely associated with all-cause mortality. However, our study was conducted among the general Japanese population and we found no significant association between green tea consumption and all-cause mortality in either males or females. A decreased risk of mortality from apoplexy was observed for men in the high green tea consumption category, but it was not statistically significant. Further studies with larger sample sizes must be conducted to define the effects of green tea on mortality.

In conclusion, habitual coffee consumption was inversely associated with death from all causes and apoplexy among men in a rural Japanese population. On the other hand, green tea consumption was not significantly associated with all-cause mortality in either men or women. The effects of habitual coffee consumption and its related factors on health in Japan need to be studied in greater detail.
